# Identification and validation of seven RNA binding protein genes as a prognostic signature in oral cavity squamous cell carcinoma

**DOI:** 10.1080/21655979.2021.1974328

**Published:** 2021-09-29

**Authors:** Zijing Huang, Tianjun Lan, Junjie Wang, Zhifeng Chen, Xiaolei Zhang

**Affiliations:** a Hospital of Stomatology, Guanghua School of Stomatology, Guangdong Province Key Laboratory of Stomatology, Sun Yat-sen University, Guangzhou, Guangdong, China; b Department of Oral and Maxillofacial Surgery, Sun Yat-sen Memorial Hospital of Sun Yat-Sen University, Guangzhou China; c Department of Stomatology, The First Affiliated Hospital of Jinan University, School of Stomatology, Jinan University, Guangzhou China; d Department of Stomatology, Nanfang Hospital, Southern Medical University, Guangzhou China; e Department of Stomatology, Linzhi People’s Hospital, Tibet China

**Keywords:** RNA-binding proteins (rbps), oral cavity squamous cell carcinoma (ocscc), overall survival, prognostic model, bioinformatics analysis

## Abstract

RNA binding proteins (RBPs) play a pivotal role in various biological processes, and aberrant expression of RBPs is closely associated with tumorigenesis and progression. However, the role of RBPs in oral cavity squamous cell carcinoma (OCSCC) is yet unveiled. In this study, RNA sequences and clinical information of OCSCC samples were acquired from The Cancer Genome Atlas (TCGA) database. A total of 650 RBPs, with significantly different expression between healthy and OCSCC samples, were identified using the limma package. A prognostic model was constructed by Lasso-Cox analysis, resulting in the determination of 7 prognosis-related RBPs: ERMP1, RNASE3, ARL4D, CSRP2, ULK1, ZC3H12D, and RPS28. Based on the prognostic model, the risk scores of the OCSCC samples were calculated. The capability of the prognostic model was further evaluated using the receiver operating characteristic curve (ROC). The areas under ROC were 0.764, 0.771, and 0.809 at 1, 3 and 5-year respectively in the TCGA dataset. Internal and external validation showed satisfactory predictive capability for prognosis in OCSCC. In addition, a nomogram was created to graphically present the model. To further validate the analytical data, qRT-PCR was performed on normal and OCSCC cell lines. The mRNA expression of the 7 prognostic genes was in accordance with the analytical results. Functional analysis and gene connection networks were used to describe the biological functions and underlying interactions among the 7 prognostic genes Overall, 7 prognosis-related RBPs were identified, which could be used to predict clinical prognosis and to identify potential therapeutic targets for OCSCC.

## Introduction

Oral cavity squamous cell carcinoma (OCSCC) is the most common malignant neoplasm of the oral cavity and causes approximately a quarter million deaths annually worldwide [1]. Due to the special locality, OCSCC progression and treatment could severely impair patients’ quality of life, including speech and swallowing functions [2]. To achieve oncologic, functional and esthetic goals, current treatment guidelines for OCSCC suggest surgery for early-stage patients and surgery along with chemoradiotherapy for advanced-stage patients [3]. Although the OCSCC treatment techniques have been improved in the past decades, the reoccurrence rate and 5-year survival rate (approximately 50%) are not yet satisfactory [1]. The risk factors for OCSCC include cigarette, alcohol, betel quid, human papillomavirus (HPV), and some genetic syndromes [4]. However, the underlying molecular mechanism and prognostic signature of OCSCC remain unclear.

RNA binding proteins (RBPs) are proteins that bind with coding and non-coding RNAs to form ribonucleoproteins (RNPs) [5]. Among human protein-coding genes, researchers have identified 1542 (7.5%) RBPs that are involved in RNA metabolism [6]. As the core of RNPs, RBPs orchestrate RNA processing and play an important role in post-transcriptional gene regulation (PTGR) – which supports cellular metabolism and coordination in RNA maturation, transportation, stabilization and degradation [5].

In recent years, many studies have suggested that the dysregulation of RBPs is responsible for a range of diseases, such as cancer and nervous and muscular system disorders [7]. Genetic knockouts of RBPs can cause death or disturb all systems, indicating the importance of RBPs in cellular processes [5]. One of the important functions of RBPs in PTGR is to modulate cell growth and proliferation. As reported by previous studies, their aberrant expression may have implications for cancer. The expression of Sam68 (a member of the signal transduction and activation of RNA metabolism family) promotes the proliferation of prostate cancer cells and resistance to cytotoxic drugs [8]. The acetylation of AGO2 supports tumor progression by promoting the biogenesis of miR-19b [9], and the overexpression of LIN28B correlates with a worse prognosis in colorectal cancer [10]. However, no literature is available no functional RBPs in OCSCC. The hypothesis of this study is that the expression level of RBPs is related to the prognosis of patients with OCSCC, and an RBP-related prognostic model could predict the prognosis of patients with OCSCC. Thus, to reveal the possible function and clinical significance of RBPs in OCSCC, bioinformatics methods were used in the current study to identify differentially expressed RBPs between healthy and OCSCC samples. Then, an RBP-related prognostic model was constructed using Lasso-Cox regression to predict the prognosis of patients with OCSCC. These findings may provide clues for prognostic signatures in OCSCC.

## Materials & Methods

### Dataset and identification of differentially expressed RBPs

In this study, RNA sequences of 16 healthy human tissues and 213 OCSCC samples with relevant clinical features were downloaded from the TCGA database by the Genomic Data Commons data portal (https://portal.gdc.cancer.gov). We then used the PERL software (https://www.perl.org) to process RNA-seq data, obtain the mRNA matrix and transform ID. The RNA-seq data used in the present study was Transcripts Per Million (TPM) format converted from Fragments Per Kilobase Million (FPKM) matrix. As an open-access database, the TCGA database consists of high-throughput sequencing and clinical information of 34 types of cancers. RBPs from *Homo sapiens* were downloaded from the EuRBPDB (http://EuRBPDB.syshospital.org) [11]. The EuRBPDB dataset includes 315,222 RBPs from 162 eukaryotic species, which enables a comprehensive analysis of the function of RBPs in various diseases.

After removed the genes with <0 expression, the limma package [12] was used to acquire differentially expressed RBPs based on a false discovery rate (FDR) < 0.05 and |log2 FC(fold-change)| > 0.5. Specifically, with the limma package, a linear model was used to analyze the expression differences of genes simultaneously.

### Construction of the prognostic model

After removing 1 OCSCC sample without complete clinical data, a total of 212 OCSCC samples from the TCGA database were included and randomly divided into a training dataset (n = 146) and an internal testing dataset (n = 66). Univariate Cox regression and Kaplan–Meier test were performed to locate prognosis-related RBPs. When the RBPs met the criteria of *P* value less than 0.05 in the above two tests, they were considered as prognosis-related RBPs. Then, these selected RBPs were collected for the least absolute shrinkage and selection operator (Lasso) and multiple Cox analysis to construct an optimal prognostic model in the training dataset. Specifically, Lasso analyses provided the optimal parameters (lambda) and identified the RBPs significantly associated with overall survival (OS) for further multiple Cox analysis to construct the prognostic model. The formula for the risk score is as follows:

Risk score = Exp_1_α_1_+ Exp_1_α_2_+ Exp_1_α_3_ + .

where *Exp* represents the expression value of the gene, and *α* represents the coefficient.

The OCSCC samples were then divided into high- and low-risk groups by comparing them to the median risk score of patients in the training dataset. The log-rank test was used to compare differences between the two subgroups. The survivalROC package was used to establish a time-dependent ROC curve for the prognostic capability of the model. Moreover, ROC analyses and multiple Cox regression analyses were performed to compare the prognostic capability between this model and traditional clinical risk factors. Univariate and multiple Cox regression analyses were used to evaluate the independent prognostic value of the regression model.

### Validation of prognostic model and construction of nomogram

In the present study, 97 cancer samples were downloaded from GSE41613 as an external testing dataset. The prognostic capability of the model was validated using the TCGA internal testing dataset and the GSE41613 external testing dataset. Log-rank test and ROC analysis were both used in the two testing datasets to evaluate the prognostic value.

Based on the above prognostic model, a nomogram – a simple graphical representation, – was used to predict 1-, 3-, and 5-year OS in the training dataset. Moreover, the calibration curves and concordance index (c-index) were used to evaluate the prediction capability of the nomogram.

### Cell culture, RNA isolation, and qRT-PCR

Human normal oral keratinocytes (NOK) was donated by Professor Jinsong Li’s lab (Sun Yat-Sen Memorial Hospital, Sun Yat-Sen University) and the human OCSCC cell line CAL33 was kindly provided by Professor Jinsong Hou (Guangdong Province Key Laboratory of Stomatology, Sun Yat-sen University). Both cell lines were cultured in DMEM (Gibco) supplemented with 10% fetal bovine serum (FBS, Gibco) and maintained at 37°C in a humidified atmosphere of 5% CO_2_.

The total RNA of NOK and CAL33 cell lines was extracted using an RNA-Quick Purification Kit (ESscience, Shanghai, China) according to the manufacturer’s protocol, and then cDNA was synthesized by reverse transcription using PrimeScriptTM RT Master Mix (Takara Bio, Ohtsu, Japan). Quantification was performed using the Hieff UNICON® Power qPCR SYBR Green Master Mix (YEASEN, Shanghai, China). The relative expression of target genes was normalized to the amount of β-actin by using the 2^−ΔΔCT^ method. The primer sequences are presented in Table 1. All results are presented as the mean ± standard deviation (SD). Comparisons between the two groups were performed using student t test with GraphPad Prism 7.0. Statistical significance was set at *P* < 0.05.

**Table 1.** Primers for qRT-PCR

Gene	Forward	Reverse
β-ACTIN	GCCGCCAGCTCACCAT	TCGTCGCCCACATAGGAATC
ERMP1	CTCTACCTGATCGCGCTGC	CTGTAGTCCTGGGGCCAATG
RNASE3	CCCACAGTTTACGAGGGCTC	ACCCGGAATCTACTCCGATGA
ARL4D	GCGGCTCACGAGAGATAAC	GGTCTTTCCAGCAGAGTCCA
CSRP2	TGGGAGGACCGTGTACCAC	CCGTAGCCTTTTGGCCCATA
ULK1	AGCACGATTTGGAGGTCGC	GCCACGATGTTTTCATGTTTCA
ZC3H12D	GCTGACACCCCTATCAGAGAG	GGTCGTCGTAGCAGACCAG
RPS28	CCGTCTGCAGCCTATCAAG	CTCGCTCTGACTCCAAAAGG

### Functional enrichment analysis and construction of correlation network

For the hub RBPs, the R package clusterProfiler 3.6.2 [13] was used to perform GO functional and KEGG pathway enrichment analysis, with *P* < 0.05. The GO analysis was comprised of biological process (BP), molecular function (MF) and cellular component (CC), and the KEGG analysis aimed to identify the functions of the genes and their related pathways. Furthermore, a correlation network was constructed to demonstrate the correlation between the hub RBPs, with a correlation value > 0.1 as the cutoff value.

### Statistical analysis

All statistical analyses mentioned in this manuscript were performed in R version 4.0.2. RBPs with FDR < 0.05 and |Log_2_FC|> 0.5 were identified as differentially expressed RBPs. The prognosis-related RBPs were identified by univariate Cox regression and Kaplan–Meier test based on criteria of *P* value less than 0.05. In qRT-PCR, the relative expression of 7 hub genes was normalized to the amount of β-actin using the 2^−ΔΔCT^ method. Comparisons between the two groups were performed by student t test with GraphPad Prism 7.0. Statistical significance was set at *P* < 0.05.

## Results

In this study, the differentially expressed RBPs in OCSCC were identified and the RBPs-related prognostic model was constructed. The GEO dataset was used to validate the performance of the model. Subsequently, qRT-PCR confirmed the mRNA expression level of RBPs in normal and OCSCC cell lines. Finally, GO and KEGG analyses revealed the biological functions and related pathways of the hub RBPs.

### Dataset and differently expressed RBPs in OCSCC

**Figure 1.**
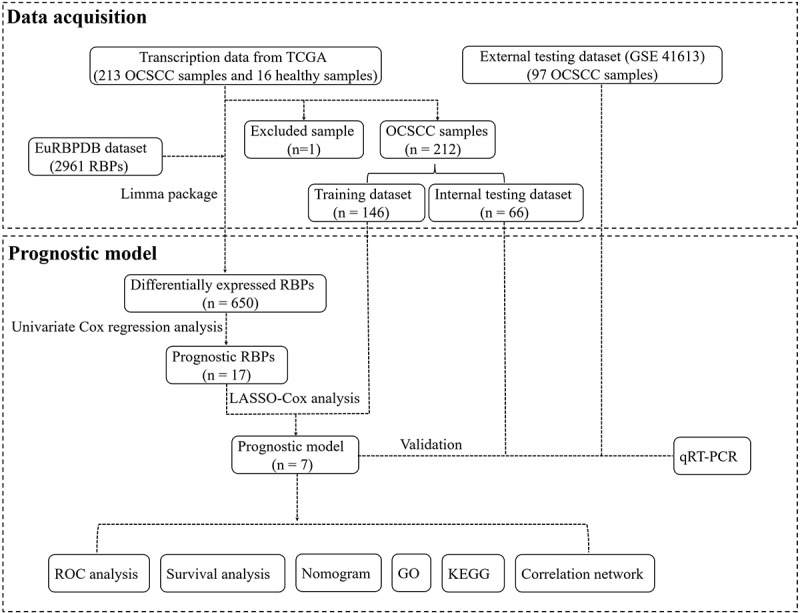
Flow chart for analyzing RBPs in OCSCC

**Figure 2.**
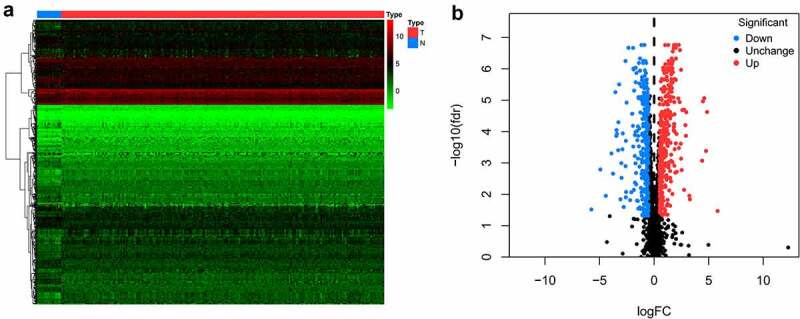
Heat map (a) and volcano plot (b) to show the differently expressed RBPs in OCSCC

A flowchart of this study is displayed in Figure 1. In this study, RNA sequences of 16 healthy tissues and 212 OCSCC samples were acquired from TCGA. Among 2961 RBPs downloaded from the EuRBPDB dataset, 650 differentially expressed RBPs were identified (*P* < 0.05, |log2FC| > 0.5), including 342 upregulated and 308 downregulated RBPs (Table S1). The heat map and volcano plot of the differently expressed RBPs are shown in Figure 2.

### Prognostic model construction

**Figure 3.**
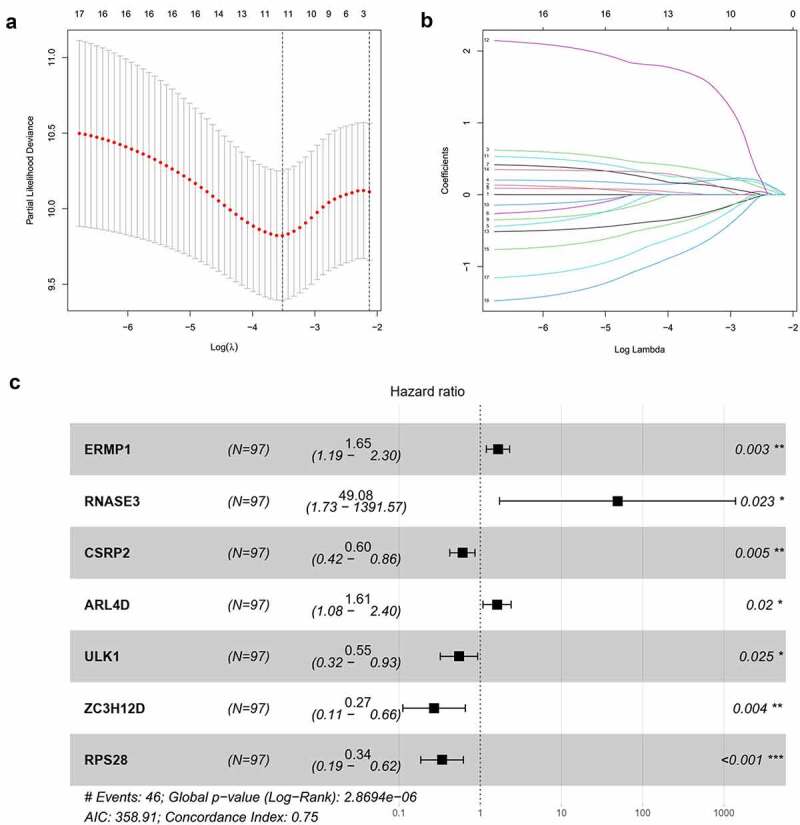
Construction of the prognostic model. (a, b) Lasso analysis identified 11 RBPs closely related to the prognosis of OCSCC. (c) Forest plot of the multiple Cox regression. Seven RBPs were determined as independent predictors for the prognosis of OCSCC

Out of 650 differentially expressed RBPs, a total of 17 RBPs were related to the prognosis of OCSCC in univariate Cox regression and K-M test (Table 2). The high expression levels of ANLN, ENO1, ERMP1, PGK1, KPNA3, GPD2, P4HA1, EGLN3, PLOD2, RACGAP1, RNASE2, RNASE3, and ARL4D were positively associated with poor prognosis. Meanwhile, the high expression of CSRP2, ULK1, ZC3H12D, and RPS28 showed the opposite tendency. Then, LASSO analysis was conducted to identify the qualified prognostic genes and eliminate overfitting of the model. These 17 differential expressed RBPs were involved in LASSO analysis and 11 RBPs were identified to be closely related to the prognosis of OCSCC (Figure 3a and 3b). To build a predictive model for time-to-event data and achieve the best prognostic performance, the multiple stepwise Cox regression was used in this study. Seven hub RBPs were finally selected to construct the prognostic model to predict the overall survival of patients with OCSCC (Figure 3c). In this model, an increased expression of ERMP1, RNASE3 and ARL4D was suggested to be related to poor prognosis, while the overexpression of CSRP2, ULK1, ZC3H12D and RPS28 resulted in a better prognosis (Figure 3c). According to this prognostic model, the risk score of patients with OCSCC was calculated using the following formula:

**Table 2.** RBPs associated with the prognosis of OCSCC in univariate Cox regression analysis

RBPs	Hazard ratio (95% CI)	*P* value
ANLN	1.362 (1.018 − 1.821)	0.037
ENO1	1.664 (1.037 − 2.671)	0.035
ERMP1	1.320 (1.061 − 1.644)	0.013
PGK1	1.978(1.311 − 2.985)	0.001
KPNA3	1.757 (1.013 − 3.045)	0.045
GPD2	1.796 (1.166 − 2.766)	0.008
P4HA1	1.374 (1.020 − 1.850)	0.037
EGLN3	1.228 (1.017 − 1.483)	0.033
PLOD2	1.301(1.027 − 1.648)	0.029
RACGAP1	1.629(1.023 − 2.595)	0.040
RNASE2	1.512(1.012 − 2.258)	0.044
RNASE3	13.051(1.977 − 86.151)	0.008
CSRP2	0.672(0.519 − 0.871)	0.003
ARL4D	1.368(1.015 − 1.846)	0.040
ULK1	0.620(0.419 − 0.917)	0.017
ZC3H12D	0.495(0.251 − 0.976)	0.042
RPS28	0.636 (0.406 − 0.997)	0.048

Risk score = (−0.5071*ExpCSRP2) + (−0.6041*ExpULK1) + (−1.3086*ExpZC3H12D) + (−1.0810*ExpRPS28) + (0.5029*ExpERMP1) + (3.8935*ExpRNASE3) + (0.4760*ExpARL4D).

**Figure 4.**
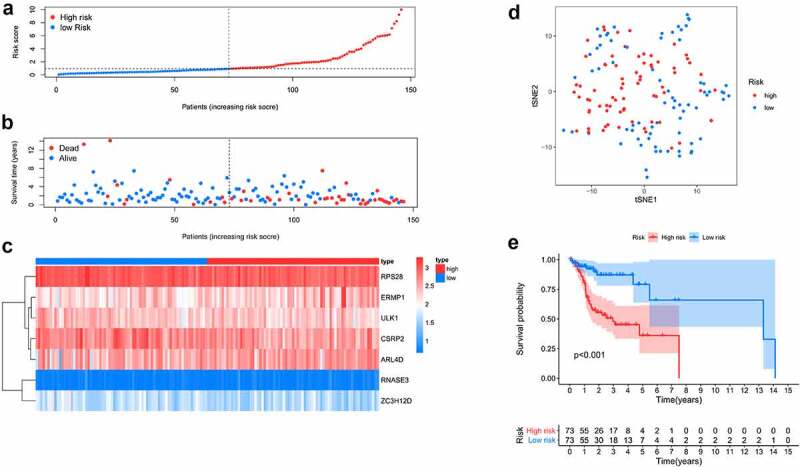
Survival analysis of TCGA training dataset. A. Dividing high and low risk group by median risk score; B. Survival time and status of OCSCC patients. C. Heatmap of the 7 significant RBPs expression. D. T-SNE analysis demonstrated that the patients in low and high risk groups were distributed in two directions. E. Survival curves showed noticeable stratification of high and low risk group

**Figure 5.**
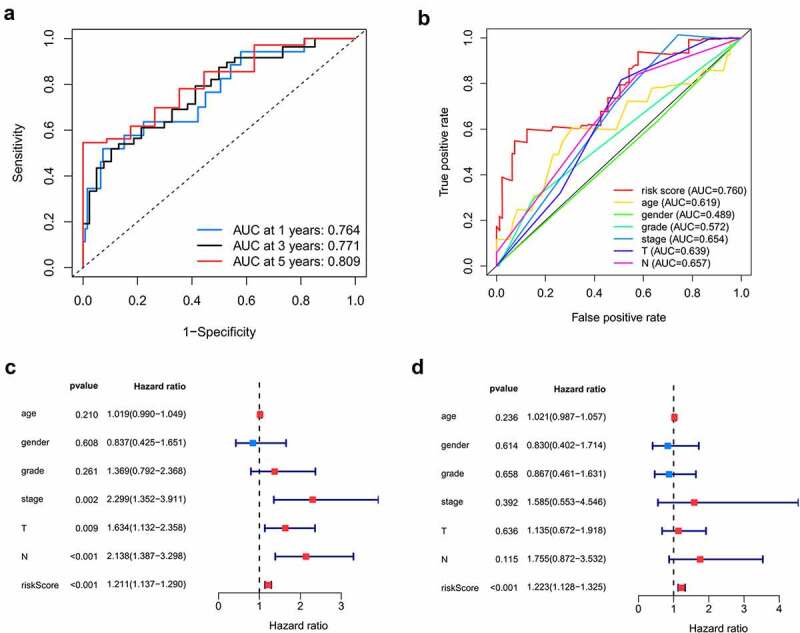
Predictive performance of the prognostic model in training dataset. A. ROC curves of the prognostic model in 1-, 3-, 5-year. B. ROC curves of the clinical risk factors and RBPs-related risk score for 1-year survival. C, D. Univariable and multiple Cox regression analysis involving clinical risk factors and the risk score

Here, 73 patients with risk scores higher than the median risk score were categorized into the high-risk group, while the other half were classified into the low-risk group (Figure 4a). As shown in Figure 4e, patients in the low-risk group had a better prognosis than those in the high-risk group. Then, t-SNE analysis were used recognize the difference of space features among samples. The patients in the low- and high-risk groups were distributed in two directions in the t-SNE analysis, indicating that these patients were well separated into low- and high-risk groups by our model (Figure 4d). ROC analysis was performed to evaluate the predictive value of the prognostic model for patients with OCSCC. The AUC of the ROC curve was 0.764 at 1 year, 0.771 at 3 years, and 0.809 at 5 years, indicating a satisfactory prediction of this prognostic model (Figure 5a). Compared to age (AUC = 0.619), gender (AUC = 0.489), grade (AUC = 0.572), stage (0.654), T-stage (AUC = 0.639) and N-stage (AUC = 0.657), our risk score demonstrated a better prediction performance with an AUC value of 0.760. Univariate Cox regression showed that the stage (HR = 2.299, 95%CI = 1.352–3.911, *P* < 0.01), T-stage (HR = 1.634, 95%CI = 1.132–2.358, *P* < 0.01), N-stage (HR = 2.138, 95%CI = 1.387–3.298, *P* < 0.001) and risk score (HR = 1.211, 95%CI = 1.137–1.290, *P* < 0.001) were associated with the prognosis of patients with OCSCC (Figure 5c). According to the multiple Cox regression analysis, the risk score remained the only independent predictor for the prognosis of patients with OCSCC (HR = 1.223, 95%CI = 1.128–1.325, *P* < 0.001) (Figure 5d).

### Validation of the prognostic model and nomogram construction

**Figure 6.**
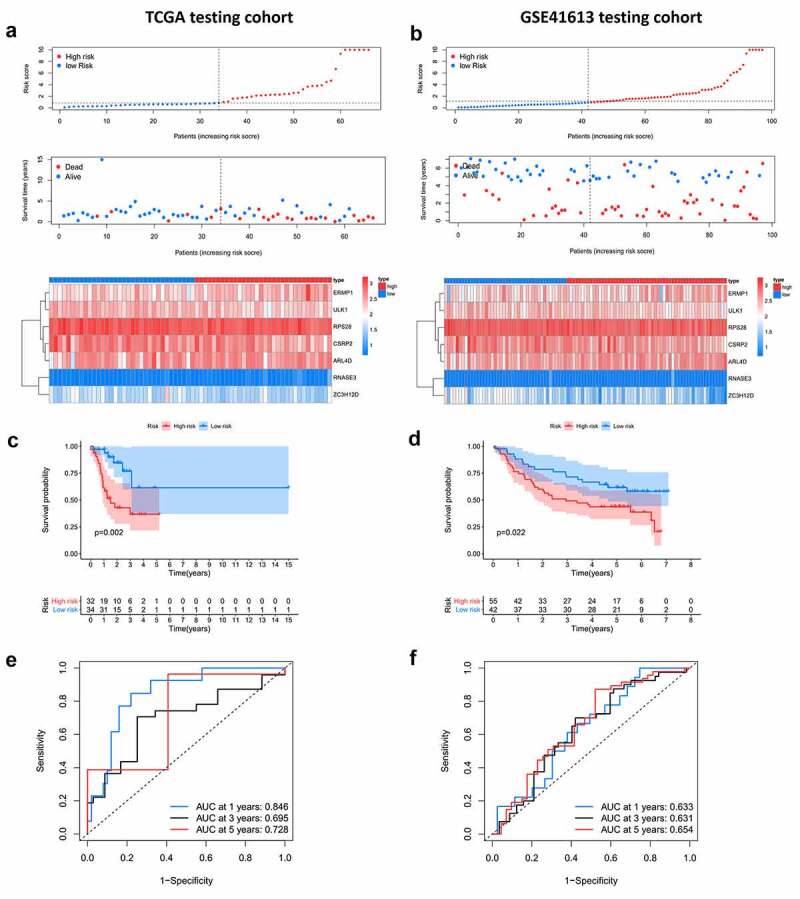
Internal and external validation of the prognostic model. A, B. The distribution of survival time, life status and the expression patterns of the 7 prognostic RBPs in TCGA internal testing dataset and GSE41613 testing dataset. C, D. Survival curves of high and low risk group showed noticeable stratification in two testing datasets. E, F. ROC curve of TCGA testing group and GSE41613 testing group in 1-, 3-, 5-year survival

The internal (TCGA) and external (GSE41613) testing datasets were used to validate the prediction of the prognostic model. The risk score formula mentioned above was used for the two testing groups. Patients in the low-risk group had a better prognosis than those in the high-risk group, which was similar to the findings in the training dataset (Figure 6a-d). The AUC of the ROC curve in the internal testing group was 0.846 at 1 year, 0.659 at 3 years, and 0.728 at 5 years, while the values in the external testing group were 0.633 at 1 year, 0.631 at 3 years, and 0.654 at 5 years (Figure 6e and f). Moreover, the present prognostic model was compared with the two published OSCC signatures [14,15]. Hou’s signature consisted of 13 prognostic autophagy-related genes, and the Miao’s model was constructed based on 7 prognostic long non-coding RNAs. Compared to the two models, our model exhibits a better performance (Figure S1).

**Figure 7.**
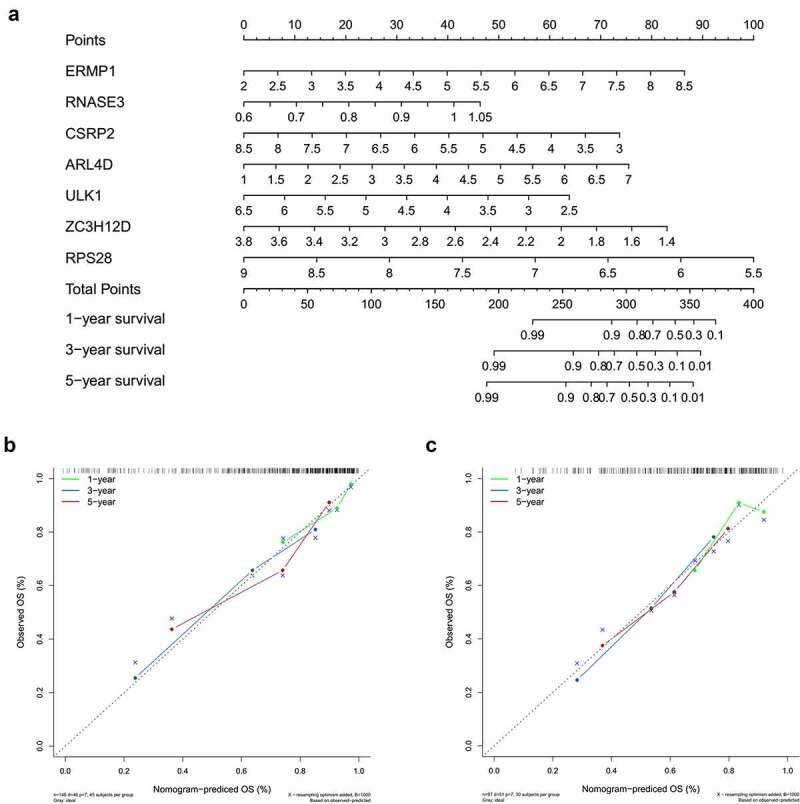
Nomogram to predict 1-, 3- and 5-year survival rate of OCSCC patients. A. Prognostic nomogram to predict survival of OCSCC patients based on TCGA dataset. B, C. The calibration plots for 1-, 3- and 5-year survival in TCGA training dataset (b) and testing dataset (c) demonstrated that the nomogram-predicted survival fit well with the actual survival

Based on the above prognostic model, the role of 7 hub RBPs was displayed in survival analysis by nomogram (Figure 7a), which was used to evaluate the prognostic prediction for patients with OCSCC. By summing up the score for each independent predictor, we obtained the total points, by estimation of the 1-, 3- and 5-year survival rates of patients with OCSCC. The predictive capability of the nomogram was validated by calibration curves and concordance index in the TCGA training dataset and external (GSE41613) testing dataset. Figure 7b shows the consistency between predicted survival and actual survival in the training dataset, with a C-index of 0.753 (95%CI = 0.714–0.792, *P* < 0.001). In the GSE41613 testing dataset, the C-index was 0.674 (95%CI = 0.670–0.678, *P* < 0.01), suggesting a decent predictive capability of the nomogram (Figure 7c).

### Validation of mRNA expression

**Figure 8.**
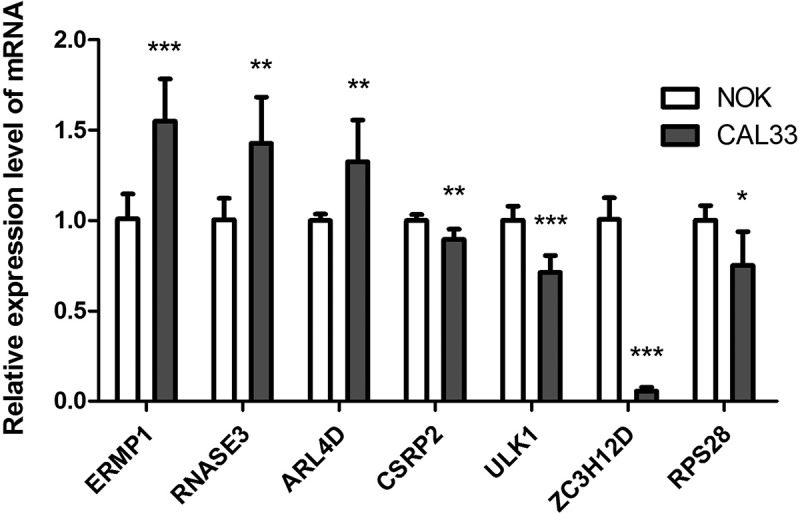
The mRNA expression level of seven prognostic genes in the normal oral keratinocyte cells (NOK) and OCSCC cells (CAL33). The expression of ERMP1, RNASE3, ARL4D were significantly upregulated in CAL33 than the control (NOK), while the expression of CSRP2, ULK, ZC3H12D, RPS28 were significantly downregulated in CAL33 compared with the NOK cells. *P < 0.05, **P < 0.01, ***P < 0.001

To further confirm the mRNA expression of RBPs in OCSCC, qRT-PCR was used to detect the mRNA expression levels of the 7 prognostic RBPs in NOK and CAL33 cell lines. In CAL33 cells, the expression of ERMP1, RNASE3 and ARL4D was significantly higher than that in NOK cells (*P* < 0.05) (Figure 8). The mRNA expression of CSRP2, ULK1, ZC3H12D and RPS28 was significantly decreased in CAL33 cells, compared with NOK cells (*P* < 0.05) (Figure 8).

### GO functional, KEGG pathway enrichment analysis and gene correlation network

**Figure 9.**
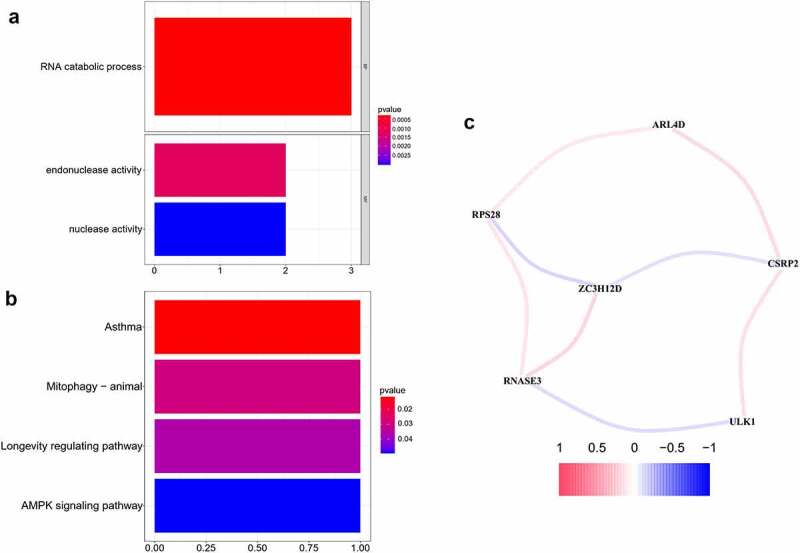
GO functional, KEGG pathway enrichment analysis and correlation network of the 7 prognostic RBPs. A, B. GO functional, KEGG pathway enrichment analysis. C. Correlation network of the 7 hub RBPs. The correlation coefficients were denoted by different colors

To further understand the biological functional pathways of the 7 hub RBPs in OCSCC, the GO functional and KEGG pathway enrichment analyses were conducted. Significant enrichment was found in the RNA catabolic process, endonuclease activity and nuclease activity (Table 1, *P* < 0.05, FDR < 0.05) (Figure 9a). Moreover, KEGG pathway analysis showed that these hub RBPs were significantly enriched in asthma, mitophagy-animal, longevity regulating pathway and AMPK signaling pathway (Figure 9b). In addition, a correlation network for the potential correlation between hub RBPs was developed. As shown in Figure 9c, the correlation network contained 8 edges and 6 nodes (ARL4D, CSRP2, RPS28, ZC3H12D, RNASE3, and ULK1). The correlation between RNASE3 and ULK1, RPS28 and ZC3H12D, CSRP2 and ZC3H12D was negative, while the other correlations were positive.

## Discussion

Despite advances in diagnosis and treatment, the outcome of OCSCC remains poor. As a malignant neoplasm, OCSCC involves the alteration of oncogenes and tumor suppressor genes. With the dawn of whole-genome sequencing, the underlying genetic changes of OCSCC were likely unveiled, which enabled early diagnosis and targeted therapy to come on the scene. Studies have shown that RBPs are crucial for post-transcriptional gene regulation [7]. Dysregulated expression of RBPs can lead to various human tumors [16]. Thus, a systematic analysis of RBP expression in OCSCC may help uncover the underlying molecular mechanism and thus identify potential diagnostic biomarkers and treatment targets for OCSCC. With the development of high-throughput techniques, researchers are able to obtain abundant online information to study using bioinformatics programs. Here, using bioinformatics resources, the RNA sequence data were acquired to analyze the expressions of different RBPs between OCSCC and healthy tissues. A prognostic model was constructed followed by an evaluation of its compatibility. Furthermore, the mRNA expression of RBPs in normal and OCSCC cell lines was assessed to validate the analytical results.

A 7-RBP prognostic model was conducted using the Lasso-Cox regression analysis. The model was validated using TCGA and GSE41613 datasets. The ROC analysis showed that the risk score obtained from the model demonstrated better prediction performance than other routine clinical factors. Currently, the TNM stage and histopathological grade are widely used by clinicians to evaluate the prognosis of OCSCC [17]. However, prognostic evaluation based on tumor stage and grade only considers tumor *per se*. According to our results, the AUC values for stage and grade were 0.654 and 0.572 respectively. Our RBP-related risk score reached an AUC value of 0.760. Our study provides a reliable way to predict the prognosis of patients with OCSCC, which could help in planning the treatment and assessing the survival.

In the present study, 7 RBPs were identified as independent predictors for the prognosis of OCSCC using Lasso-Cox regression analysis. The RBPs, including ERMP1, RNASE3, and ARL4D, were suggested to be related to poor prognosis and therefore, can be regarded as oncogenes. Endoplasmic reticulum metalloprotease 1(ERMP1) is located on chromosome 9p24 and is found to be an amplicon in cancers [18]. Qu *et al* reported that miR-148b suppresses human endometrial cancer RL95-2 cell proliferation by inhibiting ERMP1 [19]. Overexpression of ERMP1 enhances cell proliferation, whereas knockdown of ERMP1 inhibits cell proliferation. Meanwhile, ERMP1 has been found to function as a prognostic gene in kidney renal clear cell carcinoma by bioinformatics analysis, which is consistent with the findings of the present study [20]. Collectively, higher expression levels f ERMP1 may suggest a poor prognosis in cancers. RNASE3 (also known as eosinophil cationic protein, ECP) is a major eosinophil secreted protein [21]. In patients with renal cell adenocarcinoma cancer, the serum concentrations of ECP were higher than those of the controls [22]. An *in vitro* study found that ECP affected the viability of human oral squamous carcinoma cells [23]. However, considering the involvement of ECP in the immune defense processes, it is not easy to determine the role of high expression of ECP in suggesting the upcoming victory against the tumor, or the severity of the tumor [21]. In our study, the increased ECP suggested a poor prognosis of OCSCC, which was in line with the qRT-PCR results that showed higher expression of ECP (RNASE3) in oral cancer cell line. ADP-ribosylation factor (Arf)-like 4D (ARL4D) is an Arf-like small GTPase that regulates actin cytoskeleton remodeling, cell morphology and migration [24]. Lin *et al* reported that ARL4D plays an important role in microtubule growth [25]. Upregulated ARL4D promotes neurite outgrowth in N1E-115 neuroblastoma cells [26]. Taken together with our findings, ARL4D can serve as an oncogene, at least, for OCSCC.

In our study, CSRP2, ULK1, ZC3H12D and RPS28 were found to be associated with a better prognosis and can be considered as tumor suppressor genes. Cysteine-rich protein 2 (CSRP2) has been implicated in many types of cancer. In head and neck squamous cell carcinoma (HNSCC), the high expression of CSRP2 implied a better prognosis, which is highly consistent with our results [27]. A previous study reported lower expression of CSRP2 in colorectal cancer tissues than in normal tissues [28]. The progression of colorectal cancer can be suppressed by CSRP2 via ERK, PAK, and HIPPO signaling pathways *in vitro* [28]. ULK1 (unc-51 like autophagy activating kinase 1) is a key regulator of autophagy [29]. The increased acetylation of ULK1 results in the activation of autophagy in the SAS oral cancer cell line [30]. Knockout of ULK1 led to mitophagy deficiency and promoted the metastasis of breast cancer cells, which implied that ULK1 might be a therapeutic target for breast cancer. Knockdown of Aurora B promoted autophagy by decreasing mTOR/ULK1, and thereby suppressing osteosarcoma metastasis [31]. As autophagy is a double-edged sword in tumorigenesis and progression, more research is needed to elucidate the physiological and pathological functions of ULK1 in OCSCC. In this study, we found that the higher the expression of ZC3H12D (zinc finger CCCH-type containing 12D), the better the prognosis. According to the literature, ZC3H12D can regulate cell growth by binding the 3ʹ untranslated region of mRNA [32]. ZC3H12D might act as a tumor-suppressor gene by regulating cell growth in lymphoma [32] and lung cancer [33]. As for RPS28 (ribosomal proteins S28), it could influence T cell-mediated cancer immunosurveillance by regulating the generation of MHC class I peptide on the cell surface [34]. Nevertheless, RBPs in OCSCC remain to be a cap of knowledge in the literature. Considering their biological behavior and functions in different cancers, as well as the findings of the present study, it is reasonable to assume that these 7 prognostic RBPs could be used as potential biomarkers for prognosis evaluation in patients with OCSCC.

Moreover, our *in vitro* qRT-PCR analysis confirmed the differentially expressed mRNA of the 7 prognostic RBPs between normal and OCSCC cells, which served as the molecular evidence for our prognostic model. Using GO and KEGG analysis, it was found that these prognostic RBPs were involved in many biological processes and signaling pathways, for example, ULK1 participated in the AMPK signaling pathway. This could be directed toward further research on the underlying mechanisms. Nevertheless, the 7 prognostic RBPs we obtained in the present study are still poorly understood in OCSCC. Further studies are needed to elucidate their prognostic role and possible mechanisms in OCSCC.

To assess the role of prognostic RBPs and provide clinician-friendly prognostic models for patients with OCSCC, we created a nomogram based on the multiple Cox regression prognostic model. A nomogram is a prognostic device containing a simple graphical representation of a predictive model [35]. It can able to integrate biological and clinical models and has been recommended as a novel standard superior to the traditional tumor-node-metastasis (TNM) staging systems for many cancers [36].

There were some recent studied aimed to build prognostic signature for oral malignant carcinoma [14,15,37]. Zhao *et al* [37] reported a prognostic model based on GEO dataset for predicting the prognosis of oral squamous cell carcinoma. Hou *et al* [14] build an autophagy-related prognostic signature and Miao *et al* [15] constructed a lncRNAs prognostic model. However, these study lack of validation of laboratory sample. Our study provided mRNA expression validation of the 7 prognostic genes. Further molecular mechanism study of the 7 prognostic genes would be an interesting topic to explore.

Overall, our RBP-related prognostic model was built on the basis of the TCGA database and was validated in the internal (TCGA) and external (GSE41613) databases. The AUC values of the ROC curve in the training and test groups were 0.764 and 0.633, respectively, at 1 year, indicating moderate performance in predicting the overall survival of patients with OCSCC. The risk score based on our prognostic model demonstrated a better prediction performance than the current clinical factors, which further confirmed the clinical significance of the risk score in the present study. However, the construction and validation of the prognostic model were based on the existing rather than the clinical patient cohort, which was a limitation of the present study. Further prospective studies that focus on clinical patient cohorts may help to evaluate the capability of our prognostic model. The underlying biological mechanism of RBPs in OCSCC remains to be further explored.

## Conclusions

The expression of RBPs was comprehensively analyzed in OCSCC and healthy tissues. ERMP1, RNASE3, ARL4D, CSRP2, ULK1, ZC3H12D and RPS28 were associated with the overall survival in OCSCC. These findings could be used to predict clinical prognosis and to identify potential therapeutic targets for OCSCC.

## Supplementary Material

Supplemental Material

## Data Availability

All datasets used and/or analyzed during the current study are available from the corresponding author on reasonable request.

## Abbreviations

RBPs: RNA binding proteins; OCSCC: oral cavity squamous cell carcinoma; TCGA: The Cancer Genome Atlas; OS: overall survival; ROC: receiver operating characteristic curve; AUC: area under the curve; ERMP1: endoplasmic reticulum metalloprotease 1; ECP: eosinophil cationic protein; ARL4D: ADP-ribosylation factor (Arf)-like 4D; CSRP2: Cysteine-rich protein 2; ULK1: unc-51 like autophagy activating kinase 1; ZC3H12D: zinc finger CCCH-type containing 12D; RPS28: Ribosomal proteins S28; TNM stage: tumor-node-metastasis stage; SD: standard deviation

## Highlights:

(1) RNA-binding proteins participated in the prognosis of OCSCC.

(2) Seven genes were identified as prognostic biomarkers for OCSCC.

(3) Seven prognostic genes were differentially expressed between normal and OCSCC cell lines.
