# Supplementary material for: Identification and validation of seven RNA binding protein genes as a prognostic signature in oral cavity squamous cell carcinoma
Source: Bioengineered. 2021 Sep 29;12(1):7248–62. doi: 10.1080/21655979.2021.1974328 (PMC8806873; doi:10.1080/21655979.2021.1974328)
Supplement: Supplemental Material [file KBIE_A_1974328_SM9106.zip › supplementary/Supplementary Material (1).docx]

Figure S1. Comparison of RBPSig with other signatures.

Table S1. List of differentially expressed genes.
